# Supplementary material for: Impaired Mucosal Homeostasis in Short-Term Fiber Deprivation Is Due to Reduced Mucus Production Rather Than Overgrowth of Mucus-Degrading Bacteria
Source: Nutrients. 2022 Sep 15;14(18):3802. doi: 10.3390/nu14183802 (PMC9501499; doi:10.3390/nu14183802)
Supplement: Supplementary file 1 [file nutrients-14-03802-s001.zip › nutrients-1919591-supplementary.pdf]

Supplementary figures

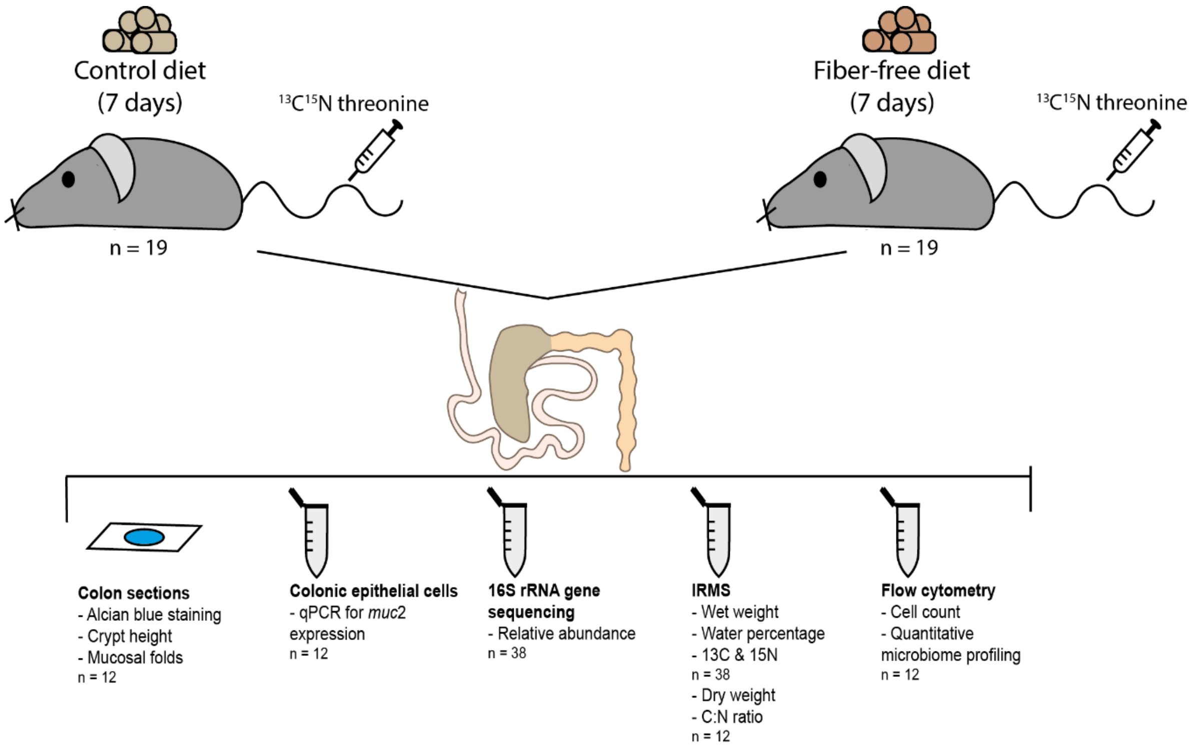

Supplementary Figure S1: Experimental set-up of harvested samples. After an initial week of acclimatization on a standard chow or control diet, half the mice were switched to a fiber-free diet. All mice were sacrificed after 7 days of dietary intervention.

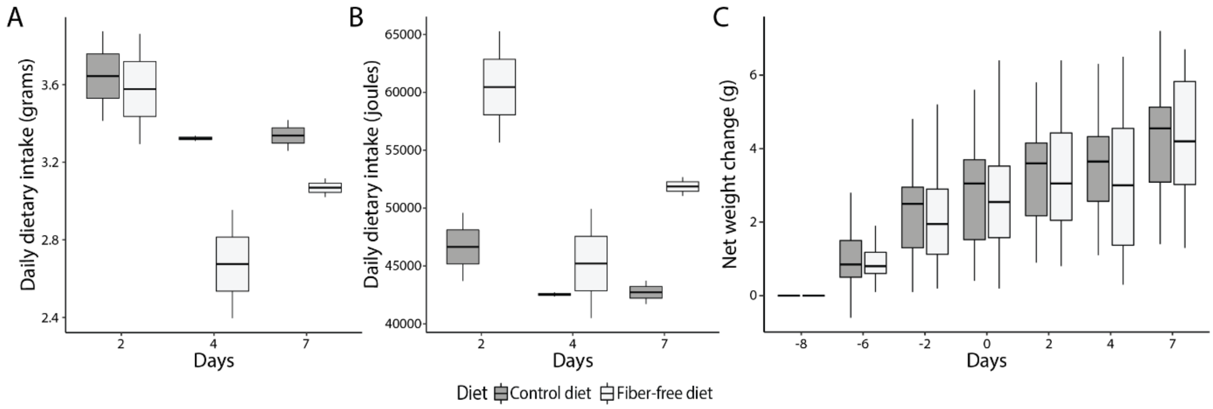

Supplementary Figure S2: Daily dietary intake in grams (**A**) and joules (**B**) per mouse per day. (**C**) The net weight change per mouse during the experiment. Day 0 indicates the day the diets were switched. Box plots show the group median and interquartile range. Dark grey indicates the control diet, light grey the fiber-free diet.

Supplementary Table S1: Nutritional composition of the diets

	Control chow diet (CD)	Fiber Free diet (FFD)
**Crude protein (%)**	19	18.5
**Crude fat (%)**	3.3	7.1
**Crude fiber (cellulose) (%)**	5 (16.9 = NDF, 7.1 = ADF)	-
**Crude ash (%)**	6.4	5.3
**Starch (%)**	35.9	-
**Sugar (Dextrose/glucose) (%)**	5.4	65.1
**Metabolisable Energy (MJ/kg)**	**13.5**	**16.9**
Protein, kcal %	33	18.3
Fat, kcal %	9	15.8
Carbohydrates, kcal %	58	65.8
